# The future of reviews: Will LLMs render them obsolete?

**DOI:** 10.1038/s44319-025-00560-z

**Published:** 2025-08-26

**Authors:** Alexander Sebastian Hauser

**Affiliations:** 1 Center for Pharmaceutical Data Science Education, University of Copenhagen, Copenhagen, Denmark; 2 Department of Drug Design and Pharmacology, University of Copenhagen, Copenhagen, Denmark

**Keywords:** Science Policy & Publishing

## Abstract

The rise of Large Language Models allows generating summaries of and analyzing the scientific literature at unprecedented speed. The academic community must acknowledge and prepare for a pivotal shift: as AI tools continue to improve, traditional literature reviews may no longer be the default approach to synthesizing research.

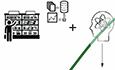

Faced with an overwhelming stack of research papers, I remember how I struggled to synthesize the available information into a coherent narrative the first time I attempted to write a review. The challenge was not just merely summarizing findings, but discerning patterns, identifying knowledge gaps and weaving a meaningful story that connects seemingly unrelated studies. This painstaking process highlights the true value of literature reviews: these are not just summaries but intellectual exercises in critical thinking and setting the stage for new insights. A good literature review also reveals limitations and blind spots in current research and provides a framework for connecting one’s findings to the existing body of knowledge.

Today, this landscape is undergoing a profound shift. Large Language Models (LLMs) such as ChatGPT, Gemini, Claude, DeepSeek, LLaMA or Mistral are increasingly used by researchers to rapidly summarize literature, explore thematic clusters, and even suggest plausible hypotheses (Lo et al, 2023; Taylor et al, 2022; Tovar, 2023). As AI tools promise to generate insights from thousands of papers in seconds, it is crucial to understand whether this evolution marks an augmentation or an obsolescence of human-driven synthesis.

As AI tools promise to generate insights from thousands of papers in seconds, it is crucial to understand whether this evolution marks an augmentation or an obsolescence of human-driven synthesis.

## The first review articles

Literature reviews in the biomedical sciences emerged very early as scholars began to distill existing medical knowledge, such as *The Hippocratic Corpus* (~400 BCE), *Galen’s Medical Compilations* (~200 CE) or William Osler’s *The Principles and Practice of Medicine* (1892). However, reviews as we know them today have only emerged during the 1930s with the founding of the *Annual Review of Biochemistry* in 1932. Faced with the overwhelming challenge of keeping up with the expanding scientific literature, J. Murray Luck, a chemistry professor at Stanford University, gathered his colleagues in 1930 to start a journal dedicated solely to synthesizing research findings. This initiative set the stage for the modern review journals that provide structured and expert-driven summaries to help navigate the growing body of knowledge.

**Figure 1:**
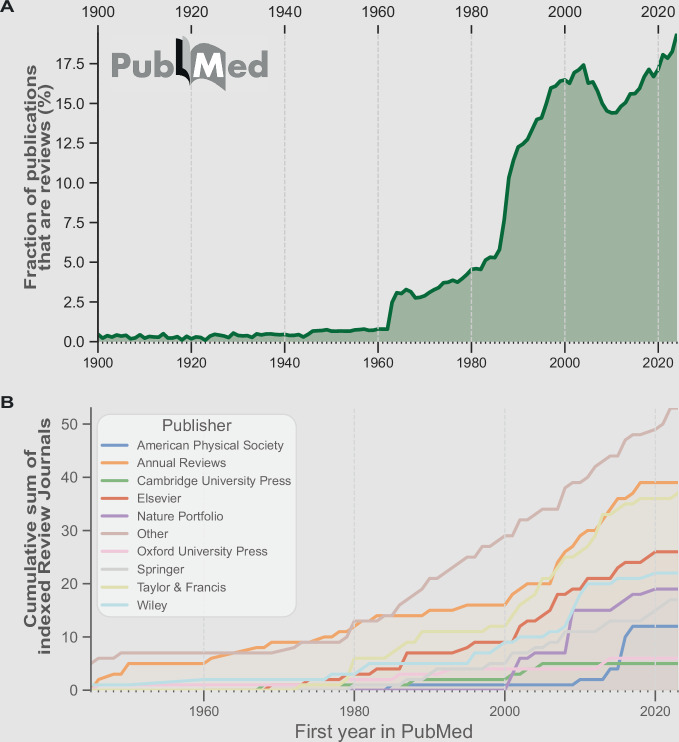
Reviews and review journals. (**A**) The fraction of published articles classified as reviews per year in PubMed from 1900 to 2024, covering approximately 40 million publication records, stratified by publication type. (**B**) Cumulative sum of journals with ‘review’ in the name among 9478 journals indexed in “Clarivate’s Science Citation Index Expanded”, grouped by publisher. First occurrence years have been obtained by querying PubMed for the first published article for each respective journal name.

The number of review articles remained relatively small, though—about 1% of the overall literature— from 1900 to the 1950s. However, starting in the 1960s and 1970s, there was a noticeable and sustained increase in the fraction of reviews among all PubMed-indexed publishing types per year (Fig. 1A). The rise of literature reviews can be attributed to the influence of Archie Cochrane and the increasing emphasis on evidence-based medicine. Cochrane’s 1972 book, *Effectiveness and Efficiency: Random Reflections on Health Services*, highlighted the need for systematic evaluations of clinical evidence, arguing that medical decisions should be guided by comprehensive and structured reviews rather than anecdotal experience. His work laid the foundation for the systematic review methodology, which would later become a cornerstone of modern medical research. According to the Cochrane Collaboration, “A systematic review attempts to identify, appraise and synthesize all the empirical evidence that meets pre-specified eligibility criteria to answer a specific research question. Researchers conducting systematic reviews use explicit, systematic methods that are selected with the aim of minimizing bias, to produce more reliable findings to inform decision making.”

**Figure 2:**
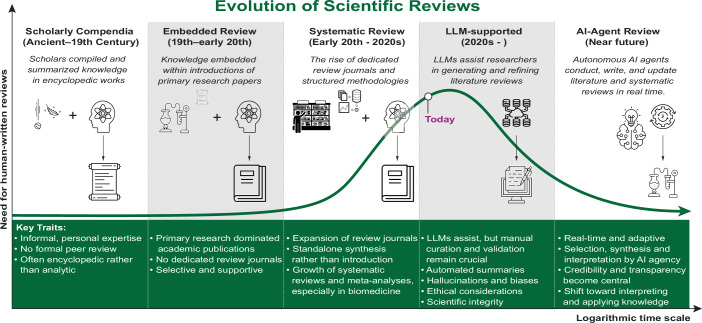
The evolution of scientific reviews. The progression of literature review methodologies and the demand for human-written reviews over time, from scholarly compendia to AI-driven review generation, following historical developments, key traits, and future perspectives. Each wave of innovation arrives faster than the last, reflecting a near-logarithmic compression of time.

After a steady rise throughout the late 20th century, the fraction of reviews saw a decline around the early 2000s, possibly caused by novel technologies and experimental techniques, such as genomics, proteomics and structural biology, which put more emphasis on original research. Around 2010, the trend reversed, and the COVID-19 pandemic further accelerated it, spurring an urgent need for rapid knowledge synthesis while limiting the capacity for primary research. Simultaneously, the rapid development of AI-driven tools and LLMs has greatly changed the mechanics of summarizing knowledge and writing literature reviews. These developments indicate that the scientific community is entering a new era in which AI-assisted reviews may become the norm, setting the stage for a transition into fully automated AI-agent literature synthesis (Fig. 2).

Today, reviews account for approximately one-fifth of all peer-reviewed publications. Following the general trend, there has been continuous growth in review journals across disciplines and publishers (Ghasemi et al, 2023), with now almost 300 dedicated review journals indexed in Web of Science’ *Science Citation Index Expanded*, underscoring their increasing role in knowledge synthesis and research dissemination (Fig. 1B).

Today, reviews account for approximately one-fifth of all peer-reviewed publications.

While this proliferation highlights the increasing role of reviews in knowledge synthesis and research dissemination, it has also raised concerns. Some argue that many of these reviews, whether systematic or narrative, offer only minimal value, are substantially redundant, contain misleading claims and/or conflicted interests (Ionnidis, 2016a). Moreover, review articles enjoy a citation premium relative to primary research papers (Ionnidis et al, 2016b) and thereby reduce citations of the original research (McMahan and McFarland, 2021), while inflating both an author’s h‑index and a journal’s impact factor. As careers, funding and University rankings are often tied directly or indirectly to these metrics, researchers, especially those in early career stages, are incentivized to write reviews. Unlike data‑heavy experimental studies, reviews require no laboratory overheads and can be produced with predictable outcomes and timelines for both authors and publishers. Commissioning reviews has therefore become a key mechanism for journals to ensure a steady stream of highly citable articles that reinforce the journal’s impact factor and authority and deliver a high return on investment for publishers.

## The rise of LLMs

The increasing role of AI in writing literature reviews introduces both opportunities and challenges. Current LLMs already accelerate the process by identifying key studies, topic filtering, and drafting an initial outline, making it significantly faster and more comprehensive (Fig. 2). This technological advancement might also improve consistency and objectivity, reducing human biases that often influence the selection and interpretation of research articles.

Several proof-of-concept systems illustrate how quickly these capabilities are maturing. A recently released AI toolset based on autonomous agents named PaperQA2, demonstrates the potential of using full-text scientific papers to generate accurate, comprehensive summaries by accessing both open-access and paywalled content (Skarlinski et al, 2024; Pearson, 2024). By digesting entire articles rather than just abstracts, these systems can identify and synthesize key information with fewer reasoning errors than traditional human-written summaries.

Beyond PaperQA2, there are other tools, such as *Scholarcy, Undermind, Amass, Elicit, Consensus* or *SciSummary*, that have been adapted to meet the needs of the scientific community: summarizing the literature, identifying argumentative gaps and valuable insights buried in otherwise unread articles, suggesting relevant studies, synthesizing contrasting scientific opinions and even conducting meta-analytic calculations (Purewal et al, 2025). These approaches require significant computational resources but provide substantially higher-quality outputs with direct literature references. LLMs yet remain unreliable at interpretive tasks such as weighing contradictory evidence, spotting methodological flaws or developing new conceptual frameworks (Skarlinski et al, 2024).

LLMs yet remain unreliable at interpretive tasks such as weighing contradictory evidence, spotting methodological flaws or developing new conceptual frameworks.

As generative models evolve in their ability to process and communicate scientific knowledge through text, a parallel transformation is occurring in the realm of visual representation. Text-to-image tools are rapidly improving and might soon be capable of generating high-quality scientific illustrations to visualize complex concepts from molecular structures to planetary systems. Generating scientific figures, however, presents unique challenges compared to natural images, as it requires representing complex relationships between discrete components often comprising a mix of plots, icons, arrows, and text. Scientific figures must also convey precise, technical information, often relying on diverse visual representations that must adhere to established conventions. To bridge this gap, new approaches focus on training generative models on large datasets of paper-figure pairs, aiming to capture the intricate relationships between textual descriptions and visual components (Rodriguez et al, 2023; Zhang et al, 2024).

## AI agents and the next leap

Looking forward, generative models driven by autonomous agents might conduct, refine and update literature information in real time, fundamentally altering the role of human researchers in systematically synthesizing scientific knowledge (Blaizot et al, 2022) (Fig. 2). By continuously integrating new findings, AI systems could address one of the key limitations of traditional reviews: their static nature. Such systems can further enhance accessibility by providing summaries tailored to diverse audiences in multiple languages or adapted to specific research questions. The emergence of AI-generated, low-cost, open-access synthesis could democratize access to cutting-edge summaries, which have historically been constrained by paywalls, disproportionately affecting researchers in low-income countries. However, this could risk shifting dependencies from traditional publishers to technology providers, with their own gatekeeping mechanisms.

By continuously integrating new findings, AI systems could address one of the key limitations of traditional reviews: their static nature.

Currently, most LLMs and generative models are limited to abstracts and publicly available articles, often excluding figures, tables and supplementary data. However, this is likely to change. Future models may gain access not just to figures, but also to the underlying datasets behind plots, raw experimental readouts, curated databases and full texts, allowing them to synthesize more detailed multimodal representations. This richer corpus would enable AI systems to generate not only more informed summaries but also data-grounded visualizations that are tailored to specific audiences or contexts. Such a foreseeable shift toward automated knowledge synthesis and presentation will likely redefine how we engage with existing scientific literature and how new insights are shared.

## A cultural shift

Literature reviews serve multiple roles beyond summarizing prior findings. Kunisch et al (2023) outline eight fundamental purposes inherent in review research. Whether explicitly stated or not, review articles contribute by classifying knowledge to establish conceptual order; representing the state of the art of a research field; problematizing existing theories and assumptions; configuring knowledge to reveal new connections; aggregating findings to support broader syntheses; integrating diverse research streams; interpreting data in a meaningful context; and explaining complex phenomena. The credibility and impact of such reviews depend on six critical aspects of rigor: design, methodological transparency and coherence; execution and application of systematic and reproducible processes; critical analysis of prior research; synthesis, developing conceptual advancements; identifying research gaps and shaping future inquiries; and fostering relevance across academic, policy and practical domains. These principles remain fundamental for human researchers even as AI-driven approaches increasingly support review processes and highlight the intellectual contribution of experts in contextualizing, critiquing and advancing scientific discourse. The current generation of LLMs and AI tools, even those specifically trained to provide summaries of the scientific literature, are not at a level that they can provide these critical aspects and principles as well as an expert in the field could do (Schwappach, 2025).

Moreover, concerns remain about scientific integrity, credibility, and ethical considerations (Liebrenz et al, 2023). While LLMs can process and summarize vast datasets, their reliance on existing literature raises the risk of reinforcing biases, misinterpreting nuanced findings or even discredited results (Gusenbauer, 2023). Moreover, LLMs are prone to generating hallucinations—fabricated or misleading information that appears plausible but lacks a factual basis—which poses a significant risk in scientific contexts (Gao et al, 2023; Tonmoy et al, 2024). Another limitation is to incorporate the latest research published after their training data cutoff, potentially leading to outdated or incomplete summaries. Overreliance on LLMs might also lead to a loss of critical human oversight. Additionally, questions about authorship, copyright and accountability in AI-generated reviews must be addressed to ensure transparency. As AI continues to evolve, striking a balance between automation and human expertise will be essential in maintaining the quality and trustworthiness of scientific literature reviews. The future belongs to those who can guide, not just gather, knowledge.

The integration of LLMs into literature reviews is therefore not a replacement for human expertise but rather a transformative shift in how knowledge is synthesized. Rather than making literature reviews obsolete, LLMs may offer an opportunity to redefine their role by enhancing the depth, accessibility and adaptability of scientific discourse. For instance, journals could adopt templates in which LLM-generated sections—for instance, summaries or background information—are explicitly tagged. Furthermore, retrieval-augmented pipelines could reduce the risk of hallucination while ensuring source traceability. That means shifting from being compilers of past research to interpreters, challengers and framers of new questions. The review of the future won’t be written about the literature; it will be written with it, in collaboration with intelligent systems.

The integration of LLMs into literature reviews is therefore not a replacement for human expertise but rather a transformative shift in how knowledge is synthesized.

If general-purpose AI systems can generate reliable, real-time, and context-aware reviews tailored to a user’s needs, it forces the next question: Do we still need a publishing system for them? This would destabilize the current ecosystem, where *authors* earn reputation by writing citable reviews, *editors* endorse quality by curating what gets published, *reviewers* filter and refine content to validate quality, and *journals* grant authority that attracts readers and justify subscriptions or open-access fees.

Much of this system is built, however, on limited expert time, limited publication space and prestige. AI-driven synthesis challenges this scarcity model by making high-quality, customized summaries abundant, on-demand and potentially free to access. In such a future landscape, the traditional roles of authors, editors and reviewers may shift from production to orchestration: guiding AI systems, verifying outputs and adding interpretive or conceptual value that machines alone cannot provide and that still require human expertise and experience. For journals, the challenge becomes existential: without the exclusivity of housing authoritative reviews, they may need to shift towards platform integration with ongoing maintenance of continuously updated knowledge hubs. In short, the question is not just whether human-written reviews will survive, but whether the socioeconomic structures that have sustained them for nearly a century can adapt to a world where synthesis is instant, ubiquitous and no longer bound to a PDF.

Beyond literature reviews, LLMs are already making significant contributions across the entire scientific process, from hypothesis discovery, virtual collaboration and experiment planning to implementation, paper writing and peer review (Luo et al, 2025; Swanson et al, 2024). To fully harness these benefits while mitigating risks, the scientific community must actively foster interdisciplinary collaboration among AI developers, domain experts, ethicists, editors and policymakers. A concerted effort is needed to align these technologies with the principles of rigorous and responsible scholarship to safeguard the integrity of scientific discourse.

Fully realizing this potential demands a transformation in how we conceptualize, publish, and interact with scientific literature. Static, monolithic review articles will give way to living knowledge bases, where AI tools continuously update the evidence in response to new findings. Reviews could evolve into modular, queryable entities, supporting ad hoc background generation, hypothesis framing, and methodological contextualization. This shift also calls for a new scholarly culture, one that values synthesis as an ongoing, collaborative practice rather than a one-off academic product. Editorial standards, peer review norms, and academic incentives must evolve to recognize and reward dynamic contributions to knowledge maintenance, not just its initial creation. This transformation will also necessitate targeted reskilling of authors, editors and reviewers with competencies in AI system evaluation, prompt engineering, and bias assessment to ensure reliability and fairness.

This shift also calls for a new scholarly culture, one that values synthesis as an ongoing, collaborative practice rather than a one-off academic product.

In this emerging paradigm, literature reviews are no longer retrospective summaries but become dynamic scaffolds for real-time reasoning across the scientific process for a faster, fairer and more connected research ecosystem. Whether society rewards these new roles will determine whether and how the review publishing system adapts or dissolves.

## Supplementary information

Peer Review File
