# Supplementary material for: The future of reviews: Will LLMs render them obsolete?
Source: EMBO Rep. 2025 Aug 26;26(18):4397–401. doi: 10.1038/s44319-025-00560-z (PMC12457580; doi:10.1038/s44319-025-00560-z)
Supplement: Supplementary file 1 — Peer Review File [file 44319_2025_560_MOESM1_ESM.pdf]

EMBO reports

TITLE: The future of reviews SUBTITLE: Will LLMs render them obsolete?

Alexander Hauser

Corresponding author: Alexander Hauser (alexander.hauser@sund.ku.dk)

Review Timeline:

Submission Date:	8th Aug 25
Accepted:	13th Aug 25

Editor: Holger Breithaupt

Transaction Report:

This article was editorially reviewed.
